# Effect of Selective Laser Melting Process Parameters on Microstructure and Properties of Co-Cr Alloy

**DOI:** 10.3390/ma11091546

**Published:** 2018-08-27

**Authors:** Jian-Hong Wang, Jie Ren, Wei Liu, Xiao-Yu Wu, Ming-Xiang Gao, Pei-Kang Bai

**Affiliations:** School of Materials Science and Engineering, North University of China, Taiyuan 030051, China; wangjianhong@nuc.edu.cn (J.-H.W.); nucrenjie@126.com (J.R.); lwnuc@163.com (W.L.); nucwuxiaoyu@126.com (X.-Y.W.); gmx252wjf@126.com (M.-X.G.)

**Keywords:** additive manufacturing, selective laser melting, CoCrMo alloy, mechanical properties, molten pool morphology

## Abstract

Due to the rapid melting and solidification mechanisms involved in selective laser melting (SLM), CoCrMo alloys fabricated by SLM differ from the cast form of the same alloy. In this study, the relationship between process parameters and the morphology and macromechanical properties of cobalt-chromium alloy micro-melting pools is discussed. By measuring the width and depth of the molten pool, a theoretical model of the molten pool is established, and the relationship between the laser power, the scanning speed, the scanning line spacing, and the morphology of the molten pool is determined. At the same time, this study discusses the relationship between laser energy and molding rate. Based on the above research, the optimal process for the laser melting of cobalt-chromium alloy in the selected area is obtained. These results will contribute to the development of biomedical CoCr alloys manufactured by SLM.

## 1. Introduction

Over the years, cobalt-chromium-molybdenum (CoCrMo) has demonstrated a remarkable level of versatility and durability as an orthopedic implant material. These alloys are widely used in biomedical applications as they are the hardest known biocompatible alloys, and also possess good tensile and fatigue properties. They were initially applied to dental implant materials, and are now commonly used in artificial hip joints and knee joints due to their excellent corrosion resistance [1,2,3] and wear resistance [4,5].

Traditional fabrication techniques such as casting [6], processes for wrought metals [7], hot pressing [1], etc. are widely used in these fields. In order to obtain good mechanical properties, scholars have conducted extensive research on the microstructure and properties of cobalt-chromium alloys manufactured by conventional techniques. For example, in order to obtain CoCrMoN alloys with good mechanical properties, an advanced refinement of grains that are similar to cast CoCr alloy can be achieved [8]. Lee studied the fracture behavior of CoCrMo alloys during tensile deformation, observing that cracks were formed at the triple junction which propagated along the annealing twin boundaries and the interface between the γ-FCC phase and the ε-HCP martensite phases [9]. Furthermore, intricate medical devices are usually difficult to fabricate using either cast or wrought CoCrMo alloys, often resulting in the waste of a high proportion of the raw materials. The problem of slow production needs to be solved and the quality of the devices improved, especially for customized medical devices.

Currently, additive manufacturing (AM) technology is being used more and more in the fields of aerospace, medical implants, and mold manufacturing. Various industries have begun to pay attention to this technology and a great deal of research, related materials, equipment, and software technology are also becoming increasingly mature. Selective laser melting (SLM) is a new additive manufacture technology that can be used for fabricating complex metallic products, such as porous structure [10,11,12,13], medical devices [14,15,16], and molds with conformal cooling channels [17,18,19]. CoCrMo alloys fabricated by SLM have been a research focus for years, because they have great potential in the manufacturing of custom artificial knee joints and oral products. Most of the previous studies have focused on the optimization of macroscopic properties, For example, the aging behavior of SLM-formed cobalt-chromium alloys. In addition, some scholars [20,21] have studied the stiffness and strength tailoring of cobalt-chromium graded cellular structures for stress-shielding reduction. However, there are few reports about the relationship between the process parameters of cobalt-chromium alloys produced by SLM and micro-melting pools. The SLM molding process is a rapid melting and rapid solidification process [22,23,24,25]. When a high-energy laser beam acts as a heat source on the surface of the metal powder, the material undergoes rapid melting and rapid solidification in a very short period of time. The rate of melting and the rate of solidification are related to the energy density of the laser irradiation. In the SLM molding process, the laser acts on the metal surface to form a molten pool. The dynamic state and thermodynamic transfer of fluid in the molten pool are the main factors affecting the geometry and internal quality of the molten pool. Selective laser melting is a process of additive manufacturing from point—line, from line—face, and from face—body. The parts are formed by layers of molten pool, so the shape and continuity of the pool determines the final performance of the part. Thus, the main aim of this study was to investigate the effects of changes in the laser melting process parameters on the morphology and macroscopic mechanical properties of the molten pool.

## 2. Materials and Methods

### 2.1. Preparation of Samples

The material used in this study was the remanium star CL model cobalt chromium alloy powder produced by the Denkurum Company (Pforzheim, Germany). The powder is a dental special material conforming to the American ISO 9693 standard [26]. It has good sphericity and can be used for laser melting in the selection area. The specific composition is shown in Table 1.

**Figure 1:**
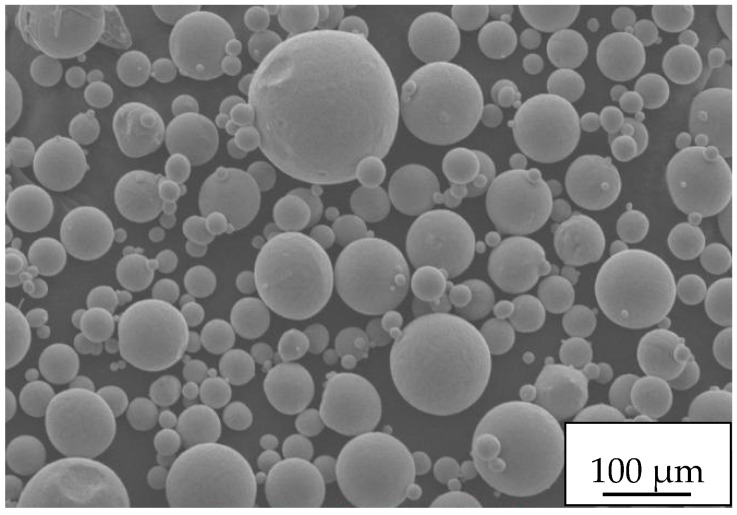
SEM of CoCr alloy powder particles.

Figure 1 shows the morphology of the powder particles under a scanning electron microscope. It can be seen that most of the powder particles have good sphericity. It can also be seen from the figure that the particle size of the powder varies, which helps to better fill the entire paving layer during the spreading.

All parts were made with the Renishaw AM 400 (Renishaw Plc., Gloucestershire, Britain). The equipment is equipped with a fiber laser with a power of 400 W. The laser model was an SDH Physical Interface (SPI) red POWER 200 W, the laser wavelength was 1071 nm, and the beam focusing diameter can reach 70 μm. The output mode was pulsed.

This study mainly discusses the effects of laser power, scanning speed, and scanning pitch on the microstructure and properties of laser-melted cobalt-chromium alloy in selected areas. The specific experimental scheme is shown in Table 2.

In Table 2, Laser Power replace with P (W), Scanning Speed replace with V(mm/s) Scanning Pitch replace with H (mm). In the Table 2, A1–A5, B1–B3, and C1–C3 are part of a single-factor experiment designed to study the relationship between laser power, scanning speed, scan line spacing, and bath morphology. Label D1-D12 comprises a multi-factor experiment designed to study the effect of process parameters on the mechanical properties and forming efficiency of cobalt-chromium alloys.

**Figure 2:**
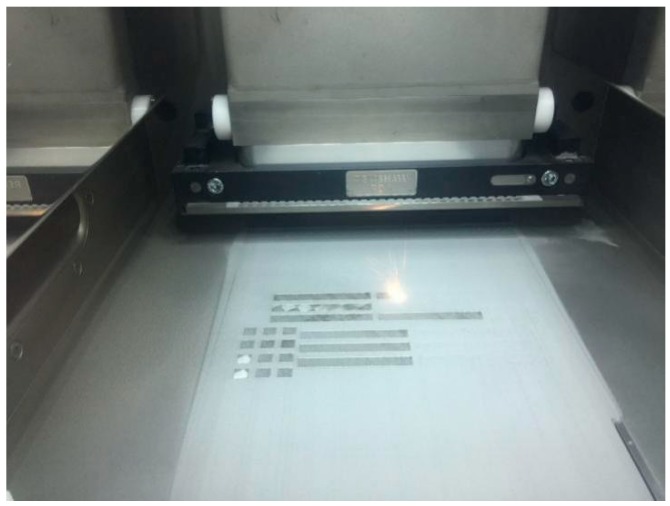
Selective laser melting of the cobalt-chromium alloy molding process.

**Figure 3:**
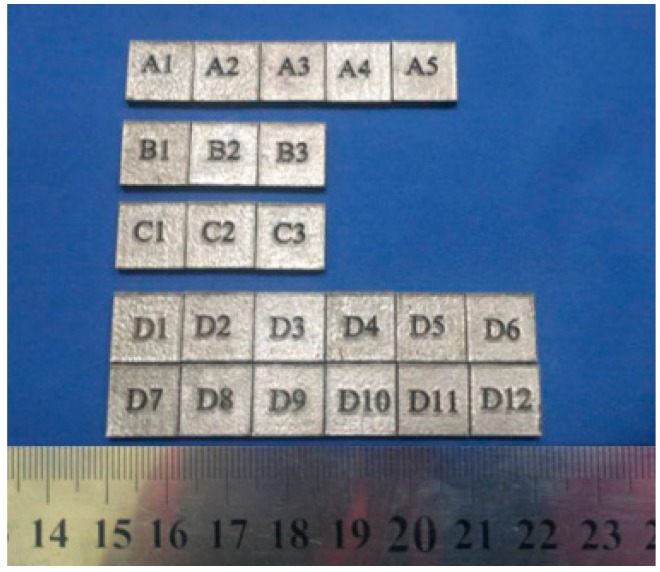
The CoCr alloy metallographic samples produced by different processes using SLM.

According to the test scheme above, the metallographic structure of the sample was made by SLM equipment, Figure 2 shows the laser melting process of cobalt-chromium alloy in a selective area, and a label was made on the top of each sample for easy distinction, as shown in Figure 3.

**Figure 4:**
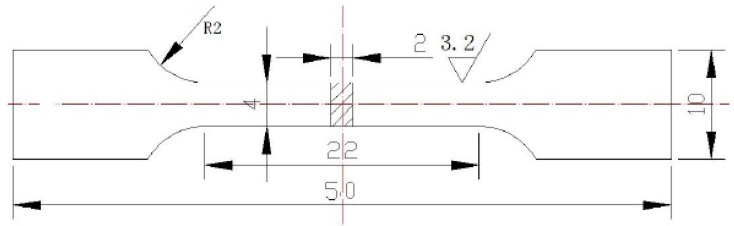
The dimensions of the stretched pattern.

Standard tensile specimens, three samples in one group, designed according to the Chinese National Standard GB/T2002 [27], were directly manufactured by SLM. The dimensions of the stretched pattern are shown in Figure 4.

### 2.2. Microstructure Observation and Analysis

The sample was polished 3000# abrasive paper, and then polished with a 2 µm Al_2_O_3_ suspension. After polishing, specimens were etched with a mixed etching agent (HCl:HNO_3_ = 3:1) for 40 s. The microstructure was observed by optical microscopy and scanning electron microscopy.

### 2.3. Mechanical Properties

A model GP-TS2000M/100kN high-temperature electronic universal tester (Shenzhen Gaopin Testing Equipment Co., Ltd., Shenzhen, China) with a displacement speed of 0.5 mm/min was used to obtain the fracture load of these specimens. All specimens were simply sand-blasted before tensile testing and stretched until they broke.

## 3. Results

### 3.1. Influence of SLM Process Parameters on the Morphology of the Molten Pool

**Figure 5:**
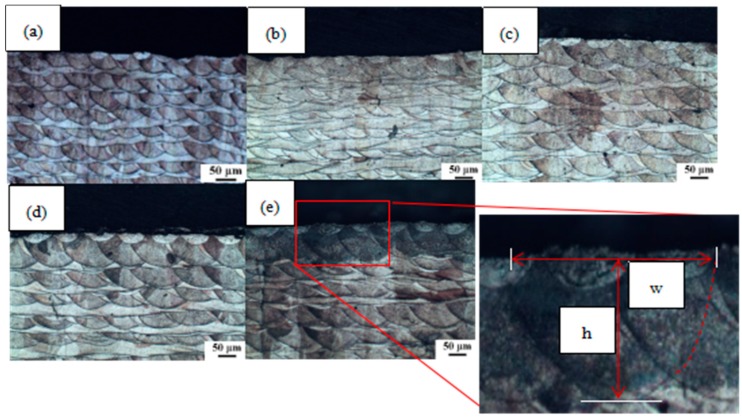
Metallographic structure of the sample at different laser powers: (**a**) 140 W; (**b**) 180 W; (**c**) 220 W; (**d**) 260 W; (**e**) 300 W.

In order to study the influence of laser power on the geometry of the molten pool, five CoCr alloy samples were prepared on SLM equipment, maintaining a scanning speed of 900 mm/s, a scan line spacing of 0.05 μm, and varying laser powers of 140 W, 180 W, 220 W, 260 W, and 300 W, respectively. Figure 5 shows the metallographic structure observed by optical microscopy after each sample was etched.

**Figure 6:**
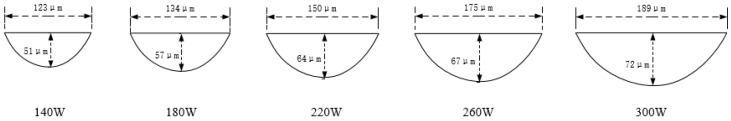
The geometry of a single molten pool at different laser powers.

We measured the specific dimensions of the 20 molten pools at the top of each sample in Figure 6 and found the average value. The width *w* and depth *h* of a single molten pool for each laser power could be obtained. The specific values are shown in Figure 6.

It can be seen from Figure 6 that as the laser power increased, more powder was melted per unit time, and the width *w* and depth *h* of the molten pool are increased to different extents [28]. *w* and *h* and the laser power were positively correlated [29,30]. It can be seen that the w and h values increased with increasing laser power, but the slope gradually decreased. It is speculated that this is because the laser energy, melt convection, and thermal diffusion increase the unusable energy, so the increased laser energy cannot be completely absorbed by the powder and the melted matrix, *w*, *h*, and laser power *P* show a positive correlation rather than an absolute proportionality [31,32,33]. The thickness of the paving layer used in this experiment was 30 μm. When the laser power was 140 W, the depth of the molten pool was 51 μm, which is larger than the thickness of the single layer and less than the thickness of the double layer. When the laser power reached 220 W, the depth of the molten pool was 64 μm, exceeding the thickness of the double layer.

**Figure 7:**
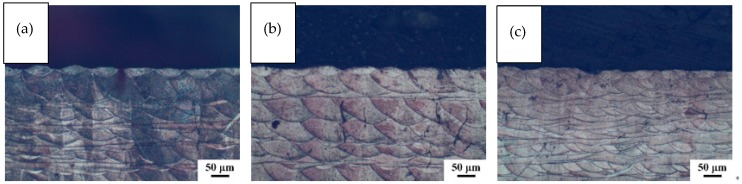
The metallographic structure of the samples at different scanning speeds: (**a**) 700 mm/s; (**b**) 900 mm/s; (**c**) 1100 mm/s.

In order to study the effect of scanning speed on the geometry of the molten pool [34], cobalt-chromium alloy samples were prepared on SLM equipment using different process parameters. The laser power, scan line spacing, and layer thickness were kept unchanged, and the scanning speed *v* was varied. A total of three speed values were selected: 700 mm/s, 900 mm/s, and 1100 mm/s. The metallographic structure of the samples at different scanning speeds is shown in Figure 7.

**Figure 8:**
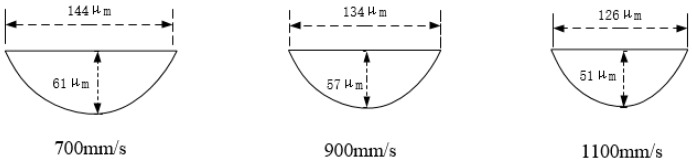
The geometry of a single molten pool at different scanning speeds.

It can be seen from Figure 7 that the cross sections of the molten pools at the three scanning speeds of this experiment were relatively regular, and there were not many defects (e.g., pores and inclusions) [35]. The slower the scanning speed, the longer the laser stays on the powder per unit area, and the more completely melted powder, as the *w* and *h* of the molten pool decrease as the scanning speed increases. If the scanning speed is too fast, the residence time of the laser per unit area of the laser per unit time is short, which will result in the metal powder not being completely melted, and the width of the molten pool is small and discontinuous. If the scanning speed is too slow, it causes the metal powder to bond and agglomerate, the spheroidization phenomenon occurs, the spheroidized protrusions may cause the surface of the molten pool to be uneven, and defects such as pores are generated, thereby affecting the effect of laying the powder on the next layer. This eventually causes a large number of defects inside the sample, and the cross section of the molten pool becomes irregular [36]. We measured the specific dimensions of the 20 molten pools at the top of each sample in Figure 7 and found the average value. The geometry of the individual molten pools at different scanning speeds could be obtained (Figure 8).

As can be seen from Figure 8, as the scanning speed increased, the laser melted less powder per unit time, and the width *w* and depth h of the molten pool decreased to different degrees, displaying a negative correlation between *w* and *h* and laser power *P* [37,38]. As can be seen, the *w* and *h* values decreased as the scan speed increased, and the slope change was small. When the scanning speed was 700 mm/s, the depth *h* of the molten pool was 61 µm, which is larger than the thickness of the two-layer layer. When the scanning speed was 1100 mm/s, the depth of the molten pool was 51 μm, which is larger than the thickness of the single layer and less than the thickness of the double layer. The greater the depth *h* of the molten pool, the greater the partially remelted portion formed, and the easier it is to form a dense structure. However, the corresponding laser utilization rate is reduced, and the forming speed of the part is also reduced.

The scan line spacing is the distance between two adjacent laser scanning trajectories, and its distance directly affects the forming quality of each layer [39]. If the scanning pitch is too small, although the tissue continuity between adjacent laser lines would be increased and the formation of pore defects would be less, the secondary remelting tends to form a coarse grain structure. This affects the performance of the product. If the scanning pitch is too large, the overlap between the two laser lines would be insufficient, and there would be no bonding between adjacent laser lines. This generates a large number of voids, thereby reducing the density.

**Figure 9:**
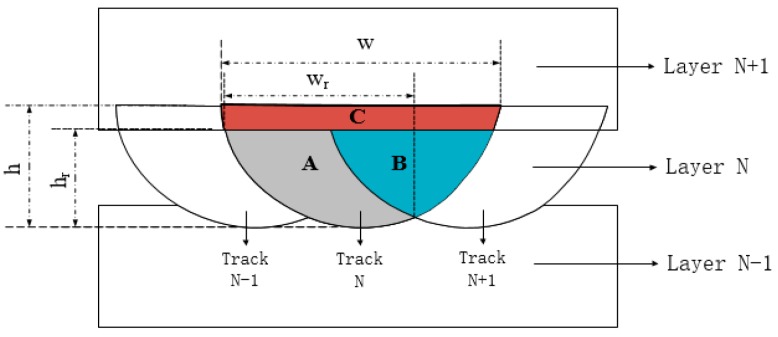
Analysis of the molten pool remelting area.

Figure 9 displays an analytical model of the remelting zone of the molten pool. The scanning strategy was to rotate 90° layer-by-layer. *w* and *h* are the width and depth of the molten pool not being remelted. *wr* and *hr* are the actual width and depth of the molten pool after remelting. The area B is a portion where the “melt pool N” is remelted by the adjacent “melt pool N + 1”. The area C is a portion in which “layer N” is remelted by the subsequent “layer N + 1”, and the area A is the actually remaining portion of “melt pool N”. Combined with previous studies, changing the laser power and scanning speed can affect the width w and depth h of the molten pool, as well as the area of regions B and C [40]. Adjusting the scan line spacing also affects areas B and C, which in turn affects the quality of the interior of the part [41].

In order to investigate the effect of the sweep line spacing on the morphology of the molten pool, cobalt-chromium alloy specimens were prepared on SLM equipment using different process parameters. We selected three scan spacing values of 0.04 mm, 0.05 mm, and 0.06 mm.

**Figure 10:**
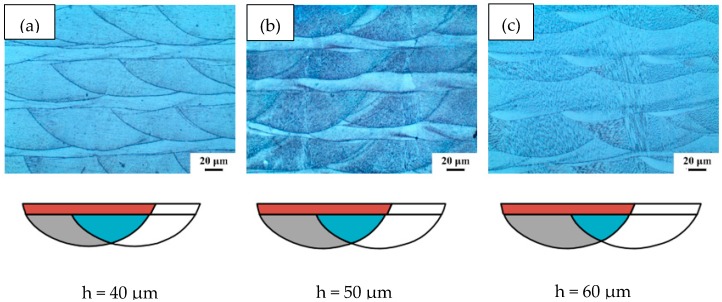
Schematic diagram of metallographic structure and molten pool overlap for different scan line spacings, (**a**) 0.04 mm; (**b**) 0.05 mm; (**c**) 0.06 mm.

Figure 10 shows a schematic diagram of the metallographic structure and molten pool overlap between different scan lines. Within the scope of this experiment, the samples under each process had relatively dense structures. Increasing the scanning line spacing increased the area of the intra-layer remelting region B and the inter-layer remelting region C of the molten pool, thereby increasing the laser utilization rate and molding rate.

### 3.2. Mechanical Properties

**Figure 11:**
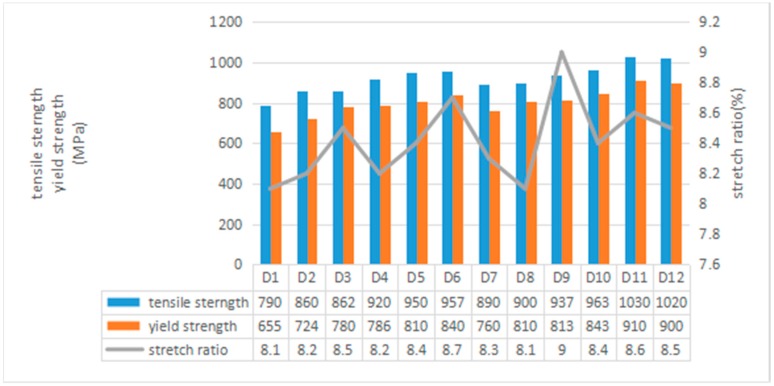
Mechanical properties of the specimens.

Tensile strength, yield strength, and elongation are typically used to characterize the tensile properties of the material. Figure 11 shows the tensile properties of SLM cobalt-chromium alloy samples prepared using the process parameters with Label D1–D12 in Table 2.

Refer to ASTM (American Society for Testing and Materials) F75 [42] for the tensile properties of cast cobalt-chromium alloys: tensile strength should be greater than 625 MPa, yield strength should be greater than 520 MPa, and elongation should be greater than 8%. It can be seen from the figure that the tensile strength and yield strength of all the samples were higher than those in ASTM F75, and the tensile strength and yield strength of sample D1 were 790 MPa and 655 MPa, respectively. This was the lowest of all samples, but still 26.4% and 25.9% higher than the standard in ASTM F75. The tensile strength and yield strength of sample D11 were the highest among all samples—1030 MPa and 910 MPa, respectively, which is 64.8% and 75% higher than the standard in ASTM F75. Parts processed using SLM technology undergo rapid melting and rapid solidification during solidification, and the grain size is much smaller than that of cast parts. The tensile strength and yield strength are higher than those of cast parts due to fine grain strengthening.

It seen that the tensile strength and yield strength of the samples increased with the increase of the laser power, decreased with the increase of the scanning speed, and increased with the decrease of the scanning pitch.

**Figure 12:**
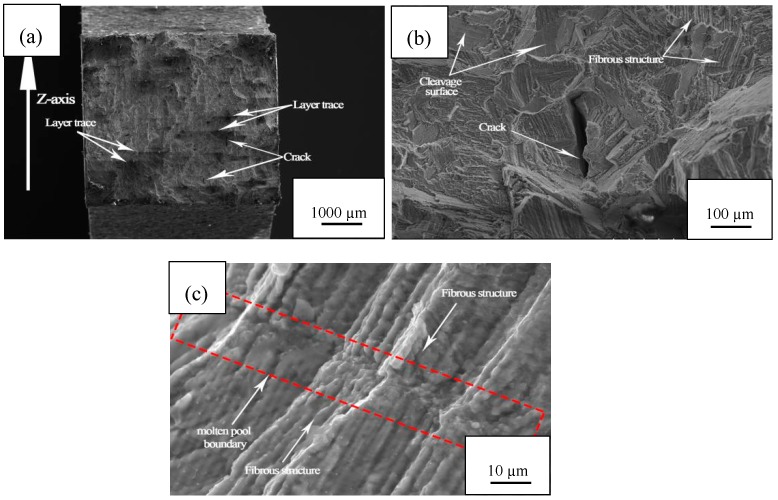
Fracture morphology of SLM cobalt-chromium alloy at different magnifications: (**a**) 1000 µm; (**b**) 100 µm; (**c**) 10 µm.

Figure 12 shows the tensile fracture morphology of sample D6. The process parameters used were: laser power P = 160 W, scanning speed v = 700 mm/s, scan line spacing = 0.04 mm. When the sample was magnified 50 times, it could be seen that there were more wedge crack marks on the cross section. When the sample was magnified 500 times, it could be found that the wedge-shaped crack had a relatively flat tearing edge, and no obvious necking phenomenon was observed around the fracture. The overall fracture mode was brittle, so it could be qualitatively considered to be a quasi-dissociation fracture. Long strips of fiber structure were observed at higher magnifications, which is very similar to the subgrain structure found in SLM cobalt-chromium alloys. Zhou [40] found that the grains having the same lattice orientation seen under Electron Backscattered Diffraction (EBSD) were actually composed of a large number of subgrains, which were elongated columnar crystal structures and had the same preferred growth orientation. The diameter of the columnar crystal was extremely small—only a few microns [43]. The long strip fibers in the observation pattern were also found to be micron in diameter and clustered to have the same orientation, and it is speculated that these fiber structures belong to subgrain structure. Furthermore, Song [44] used Electronic Differential System (EDS) to find that the columnar crystal boundary depleted the Co element enriched by the Cr element. Because the Co element accounts for about 60% in the cobalt-chromium alloy, the Cr element accounts for less than 30%. An increase in the Cr element content at the subgrain boundary causes an increase in the lattice distortion energy of the Co-based solid solution, and is more likely to cause defects such as dislocations when subjected to an external force. Therefore, when the tensile specimen breaks, cracks are generated from the subgrain boundary, and finally a fibrous morphology is left on the fracture.

### 3.3. Analysis of Molding Efficiency

In addition to density and mechanical properties, the molding efficiency of SLM technology in actual production is also an important factor to consider. The forming efficiency in the SLM process includes two concepts. The first is the forming speed, which refers to the volume of the machined part per unit time in the SLM process. The other is the laser energy density, which represents the laser energy required to melt a unit area during the SLM process. In general, the faster the molding speed, the lower the laser energy density and the higher the molding efficiency. In order to study the influence of SLM process parameters on the forming efficiency, the calculation formulas for the forming speed and laser energy density are as follows:

*ρ* = *v·h·n.* (1)

Formula (1) is the calculation formula of the forming speed, *v* (mm/s) represents the laser scanning speed; *h* (mm) represents the laser scanning line spacing; *n* (mm) represents the thickness of the paving layer; and *ρ* (mm^3^/s) represents the time it takes for the laser to melt a layer of metal powder. SLM is a layer-by-layer process with the same thickness in each layer, so *ρ* actually represents the molding speed without considering the layering time of each layer. The forming speed is proportional to the laser scanning speed and the lap joint [45].

*E* = *P*/*v·h·n* (2)

**Figure 13:**
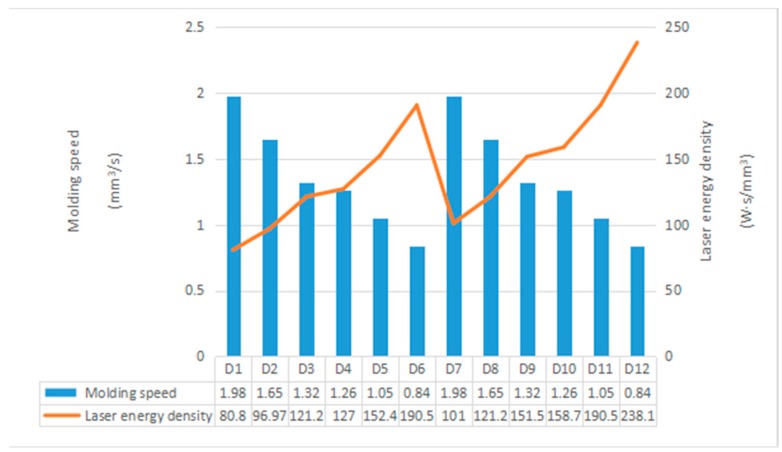
The calculation results of molding speed and laser energy density.

Formula (2) [46] is the formula for calculating the laser energy density. *P* (W) is the laser energy; *v* (mm/s) is the laser scanning speed; *h* (mm) is the laser scanning line spacing; *n* (mm) represents the thickness of the paving layer; and *E* (W·s/mm^3^) represents the laser energy consumed by melting the unit volume of metal powder per unit time. The laser energy density is proportional to the laser power, and inversely proportional to the laser scanning speed and the lap rate. The forming speed and laser energy density of the twelve process parameters in Table 2 were calculated using Equations (1) and (2). The results are shown in Figure 13.

Since the molding speed does not take the magnitude of the laser power into account, it is seen in Figure 13 that the same molding speeds were respectively obtained from D1 to D6 and from D7 to D12. Under the same process parameters, the forming speed decreased as the scan line spacing decreased (e.g., samples D1, D2, and D3). The scanning pitch was 0.06 mm, 0.05 mm, and 0.04 mm, respectively, and the forming speeds were 1.98 mm^3^/s, 1.65 mm^3^/s, and 1.32 mm^3^/s, scan line spacing of 0.04 mm and molding rate decreased by 33% compared to 0.06. As the scanning speed was reduced, the molding speed was also gradually reduced. For example, samples D1 and D4 had scanning speeds of 1100 mm/s and 700 mm/s, respectively, and the molding speed was reduced from 1.98 mm^3^/s to 1.26 mm^3^/s— a 36% reduction. Among all the samples, the highest molding speeds were D1 and D7 (also 1.98 mm^3^/s). The lowest molding speeds were D6 and D12, at 0.84 mm^3^/s. The laser energy density was opposite to the molding speed of the SLM, and is related to the laser power [47].

The order of laser energy density from high to low was: D1 > D7 > D2 > D8 > D3 > D9 > D4 > D10 > D5 > D11 > D6 > D12. The sample with the lowest laser energy density was 80.8 W·s/mm^3^ for D1, and the highest laser energy density was 238.1 W·s/mm^3^ for sample D12.

## 4. Conclusions

1. The properties of the cobalt-chromium alloy specimens were affected by the geometry of the molten pool. It was found through research and analysis that the morphology of the molten pool was related to the laser power, sweeping speed, and scanning line spacing [42]. The relationship between the micro-morphology of the molten pool and the process parameters was as follows: With the increase of laser power, the laser melted more and more powder per unit time, the width *w* and depth *h* of the molten pool increased to different degrees, and *w* and *h* were positively correlated with the laser power *P*. As the scanning speed increased, the laser melted less powder per unit time, the width *w* and depth *h* of the molten pool decreased to different extents, and *w* and *h* were negatively correlated with the scanning speed *v*. Increasing the scanning line spacing increased the area of the intra-layer remelting region B and the inter-layer remelting region C of the molten pool, thereby increasing the laser utilization rate and molding rate.
2. The tensile strength and yield strength of the sample increased with the increase of the laser power, decreased with the increase of the scanning speed, and increased with the decrease of the scanning pitch. The change in elongation of all the samples was not very different, and the regularity was not strong. The overall fracture mode was brittle, which is a quasi-dissociation fracture, and a long fiber structure similar to the subgrain structure could be observed at the fracture. By adjusting the process parameters, the properties of cobalt-chromium alloys could be changed. In this study, the influence of each process parameter on the mechanical properties of cobalt-chromium alloys was analyzed by multi-factor experiments, providing a basis for the application of SLM cobalt-chromium alloys.
3. The calculation formula of the forming speed and the laser energy density was extracted. Under the same process parameters, the forming speed was independent of the laser power, and decreased as the scanning line spacing and scanning speed decreased. The laser energy density was opposite to the law and molding speed of the SLM process parameters, and was related to the laser power. The relationship between molding efficiency and laser energy was studied in this paper. Combining the above two conclusions can achieve the purpose of ensuring both the performance of cobalt-chromium alloy and the molding efficiency.
4. According to the above three aspects, it can be concluded that the optimal SLM process parameters were: laser power 160 W, scanning speed 1100 mm/s, and scan line spacing 0.05 mm. The conclusions of this study will aid in the development of cobalt-chromium alloys for medical applications, and have great significance for the development of SLM cobalt-chromium alloys in the field of oral implants.

## Figures and Tables

**Table 1:** CoCr alloy composition.

Composition	Co	W	Si	Other Composition
Content (wt %)	60.5	9	1.5	<1

**Table 2:** The selective laser melting (SLM) test scheme.

Label	Laser Power	Scanning Speed	Scanning Pitch
	P (W)	V (mm/s)	H (mm)
A1	140	900	0.05
A2	180	900	0.05
A3	220	900	0.05
A4	260	900	0.05
A5	300	900	0.05
B1	180	700	0.05
B2	180	900	0.05
B3	180	1100	0.05
C1	180	900	0.04
C2	180	900	0.05
C3	180	900	0.06
D1	160	1100	0.06
D2	160	1100	0.05
D3	160	1100	0.04
D4	160	700	0.06
D5	160	700	0.05
D6	160	700	0.04
D7	200	1100	0.06
D8	200	1100	0.05
D9	200	1100	0.04
D10	200	700	0.06
D11	200	700	0.05
D12	200	700	0.04
